# Sequence Polymorphisms Cause Many False *cis* eQTLs

**DOI:** 10.1371/journal.pone.0000622

**Published:** 2007-07-18

**Authors:** Rudi Alberts, Peter Terpstra, Yang Li, Rainer Breitling, Jan-Peter Nap, Ritsert C. Jansen

**Affiliations:** 1 Groningen Bioinformatics Centre, Groningen Biomolecular Sciences and Biotechnology Institute, University of Groningen, Haren, The Netherlands; 2 Groningen Bioinformatics Centre, University Medical Centre Groningen, University of Groningen, Groningen, The Netherlands; 3 Bioinformatics Expertise Center, Institute for Life Science and Technology, Hanze University Groningen, University for Applied Sciences, Groningen, The Netherlands; University of Washington, United States of America

## Abstract

Many investigations have reported the successful mapping of quantitative trait loci (QTLs) for gene expression phenotypes (eQTLs). Local eQTLs, where expression phenotypes map to the genes themselves, are of especially great interest, because they are direct candidates for previously mapped physiological QTLs. Here we show that many mapped local eQTLs in genetical genomics experiments do not reflect actual expression differences caused by sequence polymorphisms in *cis*-acting factors changing mRNA levels. Instead they indicate hybridization differences caused by sequence polymorphisms in the mRNA region that is targeted by the microarray probes. Many such polymorphisms can be detected by a sensitive and novel statistical approach that takes the individual probe signals into account. Applying this approach to recent mouse and human eQTL data, we demonstrate that indeed many local eQTLs are falsely reported as “*cis*-acting” or *“cis”* and can be successfully detected and eliminated with this approach.

## INTRODUCTION

Genetical genomics–linkage and association analyses of, for example, gene expression phenotypes with the help of microarray data – is a promising strategy to identify regulatory determinants of complex traits or diseases [1]–[3]. The genetical genomics approach treats the gene expression phenotypes for each individual gene over microarrays as quantitative trait. Combined with a genetic map, quantitative trait variation can be mapped to one or more expression quantitative trait loci (eQTLs). An eQTL is said to be a local eQTL if it is on or near the genomic position of the gene, or distant if it is located elsewhere [2]. A local eQTL can include influential polymorphisms in a *cis*-acting factor, in which case it is said to be a *cis*-acting eQTL or *cis* eQTL in short. Particularly *cis* eQTLs may identify direct targets for diagnosis and treatment. It is important, therefore, that such *cis* eQTLs are identified with high accuracy and reliability. However, recent mouse studies showed that no less than 10 out of 28 genes with putative *cis* eQTLs could not be confirmed by quantitative RT-PCR [4]. Notably mRNA sequence diversity in probe regions is known to influence hybridization on microarrays considerably [5], [6]. The mRNA that is identical to the probes on the microarrays hybridizes better than the mRNA that is not identical to those probes. This causes a difference in signal between individuals with different mRNA variants, even if they have equal amounts of mRNA (gene expression). In a previous analysis we have shown how two SNPs in mouse gene ALDH9A1 caused a differential hybridization signal [7]. Here we show more examples and clearly demonstrate how in expression data from human and mouse, polymorphisms in the mRNA sequence are often falsely interpreted as *cis* eQTLs.

If a single or a few probes in a probe set distort the interpretation of the hybridization data, a statistical approach that takes the data of individual probes into account could identify and eliminate deviating probes and use the remaining probes for analysis. In a comparison of human and chimpanzee expression data, Hsieh et al. [8] eliminated probes one by one until the correlation between the profiles of the two species was >0.95. Although their method is used to eliminate deviating probes, it can not statistically test whether some probes are indeed “telling a different story”. Here we propose an extension of their method into a statistical inference procedure. We present a conceptually simple and sensitive statistical method to detect deviating and potentially problematic probes in a probe set, and we assess the utility of the new method.

## MATERIALS AND METHODS

### eQTL analysis

In this paper, a human and a mouse expression data set were analyzed. The human data set concerns samples of immortalized lymphoblastoid cells of 57 CEPH individuals hybridized to Affymetrix HG-Focus GeneChips [9] and the mouse data set concerns samples of hematopoietic stem cells of 30 BXD recombinant inbred lines hybridized to Affymetrix MG-U74Av2 GeneChips [10]. The expression data were reanalyzed using a previously reported analysis of variance (ANOVA) approach [7], here extended with a procedure to eliminate deviating probes. In short, the ANOVA model decomposes the probe signals for a given probe set into log(*y_ij_*) = *m*+*P_j_*+*A_i_*+*PA_ij_*+*e_i_*+*e_ij_*, where *y_ij_* is the hybridization signal of the *j* ^th^ probe of the *i* ^th^ sample, *m* is the average signal, *P_j_* is the average effect of the *j* ^th^ probe, *A_i_* is the average effect of the allele carried by the *i* ^th^ sample at a given genome position, *PA_ij_* is the interaction effect between probe and allele type, *e_i_* is an error term per sample and *e_ij_* is a probe-specific error term per sample. For the mouse data, additional parameters for batch effect were added (see [7]). This method is more general and flexible than the correlation approach of Hsieh et al. [8].

### Backward elimination of probes from probe sets

The ANOVA model is used to calculate the statistical significance of the interaction effects *PA_ij_*. The data on genes with distant eQTLs only, not affected by sequence diversity in the probe regions, give a good estimate of the (limited) amount of interaction present in any probe set. We therefore computed the p-values for statistical significance of the interaction terms *PA_ij_* for each of these genes in mouse, and used the 99^th^ percentile of these p-values as a threshold for genes with putative *cis* eQTLs in the human and mouse data. The procedure for the evaluation of genes with putative *cis* eQTLs starts by flagging all putative *cis* eQTLs with a significant interaction effect, *i.e.* below the threshold. Next, for all flagged genes, each individual probe is temporarily removed and the interaction effect among the remaining probes is calculated. The probe whose removal caused the largest increase in p-value of interaction effects is permanently eliminated. This procedure is repeated with the remaining probes until the p-value of interaction effects is above the threshold. The remaining probes are used for a final eQTL analysis. In cases where many probes contain SNPs, probes affected by SNPs but also probes not affected by SNPs can be considered as outliers and can be eliminated by the statistical method. To take this into consideration, all genes for which over 50% of the probes are eliminated remain flagged as having potential false *cis* eQTLs.

## RESULTS

### Human Affymetrix GeneChip arrays

**Figure 1:**
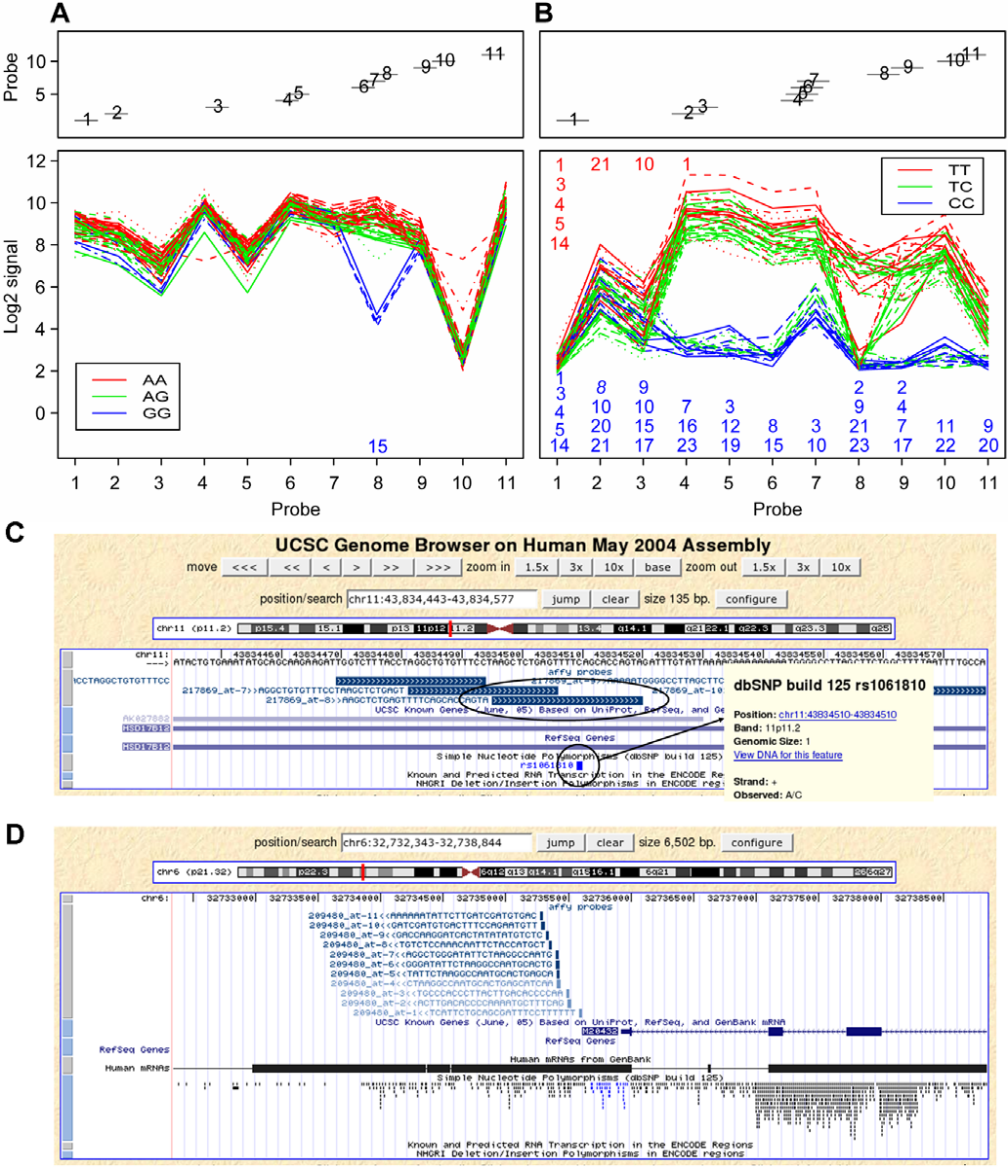
Identification of false *cis* eQTLs reported in a human association analysis. (A) Relative probe positions on the mRNA sequence (top) and hybridization signals (bottom) for gene HSD17B12 for which a *cis* eQTL is reported [9]. Each line represents one individual and is colored according to the allele that the individual carries for the associating SNP marker rs4755741. This marker is located in an intron of the HSD17B12 gene and it is strongly linked with SNP rs1061810 located in probe 8. By discarding the data for probe 8, the significance for a *cis* eQTL disappears. (B) Similar plot for gene HLA-DQB1 for which a *cis* eQTL is reported [9]. Lines are colored according to the allele the individual carries for the associated SNP marker rs6928482. One red-line and one blue-line individual have been sequenced for the probe region; the numbers in red and blue indicate the positions of SNPs within the 25-mer probe regions. The number in italic indicates a single nucleotide insertion in probe 2. We observed 23 SNPs (15 new ones) between these mRNA sequences and the 11 probes. (C) Visualization of the probes for gene HSD17B12 in the UCSC Genome Browser. Below the genomic sequence the probes are displayed in blocks. The block labels contain: probe set name, probe number, orientation on the genome (>> or <<) and probe sequence. Probe 8 and SNP rs1061810 are encircled. There is one SNP in probe 8, as was expected from probe signals. The inserted area shows information about this SNP; dbSNP shows the diversity of the SNP in the CEPH population. (D) Similar plot for gene HLA-DQB1. Probes 1-4 that do not perfectly match the genome are displayed in light blue. The current data in Genome Browser show fewer SNPs than we found in our own sequencing effort.

We simulated the occurrence of randomly distributed SNPs in Affymetrix probes using a conservative prediction of one SNP per 1000 base pairs [11]. This simulation showed that many probe sets are expected to contain one or more SNPs. For example, we predict that at least 200 probe sets of 11 probes per probe set will carry SNP variation in three or more probes on the human genome HG-U133 Plus 2.0 arrays. We also studied the distribution of known HapMap SNPs within probes; we BLASTed the Affymetrix probes of HG-U133 Plus 2.0 against the HapMap SNPs in the ten 500-kilobase ENCODE regions that were resequenced in 48 unrelated DNA samples (http://www.hapmap.org/downloads/encode1.html.en). Among the 136 probe sets present in these HapMap-ENCODE regions, 33 probe sets have SNPs in at least one probe (24%), and 5 probe sets have SNPs in three or more probes (4%). The hybridization expected to result from such variation could seriously mislead the interpretation of data from individual genes, even if only a single probe is affected. In an extensive genetical genomics experiment using Affymetrix arrays on 57 CEPH individuals, thirteen putative *cis* eQTLs were found in immortalized lymphoblastoid cells [9]. One of these eQTLs was for gene HSD17B12. Analysis of variance of the probe data of HSD17B12 with the statistical method outlined above confirms strong evidence for a local (possibly *cis*) eQTL when all probe data are used (Figure 1). However, the evidence becomes non-significant when the data of only probe 8 is left out. Inspection of the individual probe data shows that probe 8 is the only one of eleven probes that shows a clear differential hybridization signal. The Affymetrix MAS 5.0 algorithm that is routinely used to summarize probe level data is not able to single out this single probe, neither are the alternative methods dChip [12] or RMA [13]. In the CEPH individuals used, the HapMap data [11] only report an SNP (rs1061810) in probe 8, that is located at position 15 in the probe and shows A/C variation for the CEPH individuals. This SNP is strongly linked with the SNP (rs4755741) found in the association study that shows A/G variation. The data therefore indicate that the mRNA from individuals that are homozygous A for SNP rs4755741 hybridizes better than mRNA from individuals that are homozygous G for that SNP. This is as expected, because the mRNA from individuals that are homozygous A is identical to the probe sequence, whereas the mRNA from individuals that are homozygous G has a mismatch. Our procedure correctly flagged gene HSD17B12 and correctly eliminated probe 8.

A similar case is HLA-DQB1, reported as HLA-DRB2, for which a *cis* eQTL was reported in the same study [9]. We sequenced the HLA-DQB1 alleles of two individuals. Differences in the hybridization signal between these individuals could be attributed to differences between probe sequences and the actual mRNA sequences from different individuals (Figure 1). Our procedure correctly flagged gene HLA-DQB1 because more than 50% of its probes would have to be eliminated.

### Mouse Affymetrix GeneChip arrays

In an extensive genetical genomics experiment using Affymetrix arrays on 30 recombinant inbred lines in mouse, many putative *cis* eQTLs were reported [10]. Here we estimate how many of these putative *cis* eQTLs were caused by differential hybridization resulting from polymorphisms in probe regions, rather then by differential expression. We simulated the occurrence of randomly distributed SNPs in the Affymetrix mouse probes using a conservative estimate of one SNP per 1000 base pairs. This simulation confirmed that again many probe sets are expected to contain one or more SNPs. For example, we predict that at least 302 probe sets will carry SNP variation in three or more probes on the mouse genome U74Av2 arrays with 16 probes per probe set.

To study the potential influence of SNPs in probe sets on *cis* eQTL identification, we investigated the 100 most significant putative *cis* eQTLs in detail. *Cis* eQTLs can show a higher hybridization signal for the mice carrying the B6 allele (called *cis* ^B6^), or a higher hybridization signal for the mice carrying the D2 allele (called *cis* ^D2^). Because the microarray was primarily designed based on the B6 sequence, the occurrence of sequence diversity in probe regions would predict an excess of *cis* ^B6^ eQTLs. Indeed there were significantly more *cis* ^B6^ eQTLs: 70 *cis* ^B6^ vs. 30 *cis* ^D2^ (P<0.01; chi-square test). Without sequence diversity in probe regions we would expect as many *cis* ^B6^ eQTLs as *cis* ^D2^ eQTLs, that is, 30. The number of false *cis* ^B6^ eQTLs is estimated as the observed number of *cis* ^B6^ minus the expected number of *cis* ^B6^, so 70–30 = 40. This shows that almost half of the reported 100 most significant *cis* eQTLs are probably due to sequence diversity in probe regions. When applying the statistical method outlined above, 25 of the 70 *cis* ^B6^ eQTL (36%) were flagged as potentially false *cis* eQTLs. Two were flagged because more than 50% of the probes were eliminated, 22 other *cis* ^B6^ lost significance, and one *cis* ^B6^ became *cis* ^D2^. In addition, 2 of the 30 *cis* ^D2^ eQTL (7%) were flagged as potentially false for reasons that we discuss below. After the backward probe elimination procedure, (70–25 = ) 45 *cis* ^B6^ and (30–2+1 = ) 29 *cis* ^D2^ eQTLs remained, which is not a significant excess (P = 0.21; chi-square test). Our statistical method reduced the initial distortion to a non-significant level.

To assess the utility of our method, we focused on the 32 known SNPs between B6 and D2 in probes of the 100 most significant putative *cis* eQTLs. From these, 30 SNPs were derived from http://www.ncbi.nlm.nih.gov/projects/SNP/ and two additional SNPs we had identified earlier [7]. These 32 SNPs affect 25 probe sets on the microarray. Combining the hybridization signals with the SNP data, we showed that in 15 of the 25 affected probe sets the SNPs caused a difference in hybridization. Our method correctly flagged these 15 SNP-containing probe sets and successfully identified and eliminated only the SNP containing probes in those probe sets. In the remaining 10 probe sets the SNPs had no effect. This demonstrates that not every SNP causes a difference in hybridization. When the SNP is located at the very beginning or end of a probe, it can have little or even no effect on hybridization [5]. These probe sets, for which no probes needed to be eliminated, correctly remained unflagged.

Most probes on the mouse array are based on the B6 sequence, but not all. It is therefore possible that a deletion or insertion in the B6 sequence and/or an SNP between the B6 sequence and the probe sequences can cause false *cis* ^D2^ eQTLs. Indeed, one of the two *cis* ^D2^ eQTLs that were flagged was found to be false: it was caused by a combination of a known deletion and an SNP in the B6 sequence of gene H2-D1 in comparison to the probe sequences.

## DISCUSSION

In genetical genomics experiments, putative *cis* eQTLs are thought to often reflect differential gene expression between individuals [1]–[4]. However, it is shown here that in many cases such putative *cis* eQTLs should be considered with extra care: the data that are interpreted as reflecting differential gene expression could actually be due to sequence diversity in the probe regions. Without expression differences, such erroneous *cis* eQTLs will not be targets for diagnosis and treatment.

Our analysis of short-oligomer data from human and mouse Affymetrix microarrays demonstrates that the issue of differential hybridization due to sequence diversity in probe regions is systematic. In the mouse data, it caused an excess of *cis* ^B6^ eQTLs, because the arrays were designed using sequences of the B6 parental line. In a recent rat study [14] probes were mainly based on ESTs and cDNA sequences from outbred animals and not from one of the parental strains used to generate the segregating population. Therefore no excess was observed, but this absence of an excess does not mean that the data do not suffer from sequence diversity in probe regions.

The occurrence of differential hybridization due to sequence diversity in probe regions may be thought specific for short-oligomer arrays: sequence differences in short sequences are supposed to affect hybridization more than in longer sequences. However, we also used 60-mer cDNA microarrays with one probe per gene, designed on the basis of the sequence of the N2 strain of *Caenorhabditis elegans* [15]. Among the 100 most significant putative *cis* eQTLs there was an excess of 74 *cis* ^N2^ eQTLs (enrichment significant at P<<0.001; chi-square test). This may indicate that sequence diversity in probe regions can also result in false *cis* eQTLs in case of long-oligomer microarrays.

To properly deal with the issue of differential hybridization due to sequence diversity in probe regions, we recommend using multiple (tiling) probes per gene that allow statistical filtering as developed in this paper. Three metrics were helpful to assess the utility of the statistical method in a set of 100 most significant putative *cis* eQTLs in a mouse study:

1. Is the statistical method flagging as many genes with *cis* ^B6^ eQTL as the observed excess of *cis* ^B6^ over *cis* ^D2^ eQTLs? The excess is estimated to be 40, of which 25 are flagged. This could suggest that 15 false positive *cis* ^B6^ eQTLs go unnoticed. The power would then be approximately 63%. However, after applying our procedure the remaining unflagged genes do not show a significant excess and this deviation from equal proportions could just as well reflect random variation. That implies that the power can be much higher than 63%. If wished, we could increase the power by changing the settings of the elimination procedure to flag more genes with *cis* ^B6^ eQTLs (possibly leading to more false negatives). Peirce et al. [16] suggest that other biological phenomena, for example directed loss of DNA in D2 relative to B6, could explain some excess of *cis* ^B6^ eQTLs.
2. Is the statistical method flagging only genes with *cis* ^B6^ eQTLs and no genes with *cis* ^D2^ eQTLs? In addition to 25 genes with *cis* ^B6^ eQTLs, two genes with *cis* ^D2^ eQTLs were flagged. One of these *cis* ^D2^ eQTLs was due to a deletion and SNP, *i.e.* the gene was correctly flagged. The enrichment of 25 *cis* ^B6^ over 1 *cis* ^D2^ is striking. Moreover, it cannot be excluded that also the second gene with *cis* ^D2^ eQTL is flagged correctly. These results strongly suggest that the method is very specifically flagging a *cis* ^B6^ related phenomenon without erroneous rejections.
3. Is the statistical method flagging all probe sets carrying known SNPs? Indeed, the method correctly flagged all 15 SNP-containing probe sets with influential sequence diversity in probe regions, successfully eliminated only the SNP containing probes in those probe sets, and identified 100% of the false *cis* eQTLs. Probe sets with known SNPs with no effect on hybridization were not affected by the method (no probes eliminated; gene not flagged). Amongst the 25 flagged genes with *cis* ^B6^ eQTLs, 15 carry known SNPs, but the remaining 10 do not. This could suggest 40% erroneous rejections of true *cis* eQTLs but, in combination with (ii) above, more likely reflects the (still) incomplete information on SNPs or other forms of polymorphisms, such as insertions and deletions between B6 and D2.

Obviously statistics alone can not solve what is essentially a biological phenomenon: sequence diversity in probe regions between individuals. For this reason we strongly recommend that additional genome-wide methods to characterize polymorphisms [17], re-sequencing of probe regions, and alternative ways of gene expression profiling are employed whenever strong claims about *cis* eQTLs are to be made. Only then large-scale mapping of the determinants of gene expression will become truly informative.
